# Supplementary material for: WordCommentsAnalyzer: A windows software tool for qualitative research
Source: F1000Res. 2018 Sep 4;7:536. Originally published 2018 May 3. [Version 2] doi: 10.12688/f1000research.14819.2 (PMC6137411; doi:10.12688/f1000research.14819.2)
Supplement: Supplementary file 3 [file f1000research-7-17614-s0002.tgz › b2a79496-4ada-47c4-9682-a99ce867836d.zip!/Eight Iranian Health Journals/Journal of Knowledge and Health_1.docx]

© Journal of Knowledge and Health. This work is licensed under CC BY 4.0 (https://creativecommons.org/licenses/by/4.0/).

Volume & Issue: دوره 13، شماره 1397:1

Title: بررسي نقش پيش‌بيني‌‌كنندگي تاب‌آوري، حمايت اجتماعي و هوش معنوي در بهزيستي فاعلي مادران داراي دانش‌آموزان كم‌توان ذهني

http://knh.shmu.ac.ir/index.php/site/article/view/1889

مقدمه: باتوجه به اثرات مثبت تاب‌آوري، حمايت اجتماعي و هوش معنوي بر انطباق مثبت و رشد فرد در شرایط مشکل، هدف از اجراي این پژوهش بررسی نقش پیش‌بینی‌کنندگی تاب‌آوري، حمایت اجتماعی و هوش معنوي در بهزيستي فاعلي مادران داراي دانش‌آموزان كم‌توان ذهني است. مواد و روش‌ها: اين پژوهش يك مطالعه توصیفی از نوع همبستگی است كه جامعه آماری آن، کليه مادران (436 نفر) دانش‌آموزان کم‌توان‌ذهنی آموزش‌پذیر مشغول به تحصیل در مدارس استثنایی شهر اراک در سال تحصیلی 96-1395 بود. بدين منظور، 216 نفر از مادران مدرسه استثنایی نور در شهر اراک به روش نمونه‌گیری در دسترس از جامعه انتخاب شده‌‌اند. ابزارهای گردآوری اطلاعات، شامل پرسشنامه حمایت اجتماعی واکس، مقياس سنجش هوش معنوي کینگ، مقياس تاب‌آوري کانر و دیویدسون، مقياس شادکامی فاعلي ليوبومرسکي و لپر و مقياس رضايت از زندگي دينر بودند. برای تجزیه‌وتحلیل داده‌ها از ضريب همبستگي پيرسون و تحليل رگرسيون چندگانه استفاده شد. نتایج: نتایج همبستگی پیرسون نشان داد که تاب‌آوري، حمايت اجتماعي و هوش معنوي با بهزيستي فاعلي و ابعاد آن (شادكامي و رضايت از زندگي) رابطه مثبت معنی‌دار دارد. همچنين نتايج رگرسيون چندگانه نشان داد كه هوش معنوي (42/0=B)، حمايت اجتماعي (37/0=B) و تاب‌آوري (11/0=B) می‌تواند تغییرات مربوط‌به بهزيستي فاعلي را در مادران دانش‌آموزان کم توان ذهنی، به‌صورت معنی‌داری پیش‌بینی کند و 43 درصد از واریانس بهزيستي فاعلي توسط این متغیرها تبیین می‌شود.نتیجه‌گیری: با افزایش هوش معنوي، حمایت اجتماعی و تاب‌آوری می‌توان بهزیستی فاعلي (شادكامي و رضايت از زندگي) را در مادران افزایش داد.

Title: بررسی اثر استرس شغلی بر فرسودگی شغلی در میان پرستاران

http://knh.shmu.ac.ir/index.php/site/article/view/1758

مقدمه: فرسودگی شغلی از مهمترین عوامل تأثیرگذار بر روی تمایل به ترک شغل در میان پرستاران و کیفیت ارایه خدمات مراقبت‌های بهداشتی است. هدف از مطالعه حاضر بررسی اثر متغیرهای دموگرافیک، سازمانی و ابعاد مختلف استرس شغلی بر روی فرسودگی شغلی در میان پرستاران می‌باشد. مواد و روش‌ها: این مطالعه مقطعی برروی 170 نفر از پرستاران یکی از بیمارستان‌های شهر شاهرود، که به‌صورت تصادفی انتخاب شدند، انجام گردید. داده‌های با استفاده از پرسشنامه‌های دموگرافیک، استرس شغلی پرستاران (NSS) و فرسودگی شغلی مسلش (MBI) جمع‌آوری گردید. تجزیه و تحلیل داده‌ها با استفاده از آزمون‌های ضریب همبستگی پیرسون و رگرسیون لجستیک چند متغیره و نرم‌افزار SPSS  در سطح معنی‌داری 05/0 انجام شد. نتايج: از میان ابعاد استرس شغلی تعارض با سایر همکاران و عدم حمایت به‌ترتیب دارای بیشترین (45/4±79/11) و کمترین (64/1±77/3) میانگین بودند. بیشترین میانگین فرسودگی شغلی مربوط‌به خستگی عاطفی (65/9 ±29/19) بود. خستگی عاطفی با کمبود حمایت (18/3–14/1=CI، =OR1/91)، عدم آمادگی کافی (57/3–13/1=CI، =OR2/01)، شیفت کاری (988/0–046/0=CI، =OR 0/214) و درگیري با پزشکان (92/1–45/1=CI، =OR1/64) رابطه معنی‌دار داشت. علاوه‌بر این، مسخ شخصیت با مرگ و مردن و کمبود حمایت، رابطه معنی‌دار مثبت و موفقیت فردی به‌ترتیب با عدم آمادگی کافی رابطه معنی‌دار منفی و با سابقه کار رابطه معنی‌دار مثبت داشت. نتیجه‌گیری: نتایج مطالعه حاضر نشان داد که با ارتقاء برخی از ابعاد استرس شغلی مانند تعارض با پزشکان، عدم‌آمادگی کافی، کمبود حمایت، مرگ و مردن و همچنین بهبود نظام نوبت‌کاری می‌توان باعث کاهش فرسودگی شغلی پرستاران شد.

Title: بررسي رابطه اختلال خواب و كيفيت زندگي جنسي در پرستاران شيفت در گردش

http://knh.shmu.ac.ir/index.php/site/article/view/1869

مقدمه: پرستاران به‌دليل اشتغال در نوبت كاري شب دارای مشکلات خواب می‌باشند. کیفیت زندگی جنسی می‌تواند تابلویی از وضعیت سلامت و کیفیت عمومی زندگی افراد باشد. هدف اين مطالعه بررسي ارتباط كيفيت خواب و كيفيت زندگي جنسي در پرستاران زن می‌باشد. مواد و روش‌ها: مطالعه حاضر يك مطالعه مقطعي بوده و جامعه مورد مطالعه، پرستاران زن شاغل در بیمارستان‌های دانشگاه علوم پزشکی شاهرود (امام حسين و بهار) می‌باشد. نمونه‌گيري به‌صورت نمونه‌گیری در دسترس انجام گرفت. معیارهای ورود به مطالعه عبارتند از: سابقه يك سال كار به‌صورت شيفت در گردش در بيمارستان، داشتن حداقل دو شیفت شب در هفته، متأهل بودن و زندگی با همسر و تمایل به شرکت در مطالعه. همه شرکت‌کنندگان فرم رضـایت آگاهانـه را پیش از ورود به مطالعه تکمیل نمودند. ابزار گردآوری داده‌ها عبارت بود از فرم اطلاعات دموگرافیگ و تاريخچه پزشكي، پرسشنامه کیفیت زندگی جنسی زنان، پرسشنامه خواب پيتزبورگ و پرسشنامه خودکارآمدی جنسی. براي بررسی ارتباط بین عوامل دموگرافیک، تاريخچه پزشكي و کیفیت زندگی جنسی با خودکارآمدی جنسی از آزمون آماری تجزیه و تحلیل واریانس (ANOVA) و آزمون تی مستقلsample t-test) )Independent استفاده شد. براي تعیین عوامل پیش‌بینی‌کننده وضعیت کیفیت زندگی جنسی واحدهاي تحت پژوهش از تجزیه و تحلیل رگرسیون خطی چندگانه استفاده گردید. نتایج: از بين 285 پرستار واجدشرايط، پرسشنامه 260 نفر براي تجزیه و تحلیل نهايي مورد ارزيابي قرار گرفت. نتايج بررسي نشان داد كـه 4/65 % از شركت‌كنندگان داراي كيفيت خواب در سطح ضعيف هستند. ميانگين نمره كيفيت زندگی جنسي نیز 71/8±60/67 به‌دست آمد. در بين متغيرهاي مورد مطالعه، سن پرستاران، سن همسر، مدت ازدواج، سابقه کار، خودکارآمدی جنسی و کیفیت خواب، همبستگي معني‌دار مستقیم با کیفیت زندگی جنسی نشان داد. نتیجه‌گیری: باتوجه به اینکه متغیرهاي مورد بررسی 44 درصد تغییرات در کیفیت زندگی جنسی را تبیین می‌کنند و مهم‌ترين عامل پيشگويي‌كننده كيفيت خواب به‌دست آمد با در نظر گرفتن این نتایج می‌توان به ارتقاء کیفیت زندگی جنسی مبادرت نمود.

Title: پاسخ متابولیکی استخوان به فعالیت‌های ورزشی هوازی در مردان سالم کم‌تحرک

http://knh.shmu.ac.ir/index.php/site/article/view/1907

مقدمه: هدف از اجرای این مطالعه بررسی پاسخ مارکرهای بیوشیمیایی متابولیسم استخوان به فعالیت‌های ورزشی هوازی در مردان سالم کم‌تحرک بود. مواد و روش‌ها: در این مطالعه نیمه‌تجربی، 22 مرد سالم کم‌تحرک با میانگین سنی 1/3±2/35 سال در دو گروه تجربي (12 نفر) و کنترل (10 نفر) قرار گرفتند. آزمودنی‌های گروه تجربی به مدت 12 هفته در یک برنامه تمرينات هوازي شرکت نمودند. گروه کنترل در هیچگونه فعالیت ورزشی شرکت نداشتند. ظرفیت هوازی، درصد چربی بدن و سطوح سرمی کربوکسی ترمینال تلوپپتید کلاژن نوع یک و آلکالین فسفاتاز قبل و بعد از دوره تمرين اندازه‌گیری شد. برای تجزیه و تحلیل داده‌ها از آزمون تی زوجی و تی مستقل استفاده شد. نتایج: پس از تمرینات هوازی تفاوت معنی‌داری در سطوح کربوکسی ترمینال تلوپپتید کلاژن نوع یک و آلکالین فسفاتاز بین دو گروه ایجاد نشد اما ظرفیت هوازی و درصد چربی بدن آزمودنی‌های گروه تجربی به‌طور معنی‌داری بهبود یافت.نتیجه‌گیری: یافته‌های این مطالعه نشان داد که 12 هفته تمرینات هوازی بر مارکرهای بیوشیمیایی متابولیسم استخوان مردان کم‌تحرک تأثیری نداشت. احتمالاً شدت تمرینات به‌کار رفته برای ایجاد تغییرات مارکرهای متابولیکی استخوان کافی نبوده است.

Title: اثر یک دوره خود تزریقی سوستانون و مصرف امگا 3 بر سطوح سرمی کلسترول تام، عامل مغذی عصبی مشتق از مغز، لیپوپروتئین پر چگال، لیپوپروتئین کم چگال و آلانین آمینو ترانسفراز در بدنسازان مرد

http://knh.shmu.ac.ir/index.php/site/article/view/1912

مقدمه: اثرات نامطلوب استفاده نادرست از داروهای آنابولیک-آندروژنیک شامل اختلالات قلبی-عروقی، سوء عملکرد کبد، بیماری کلیه، مشکلات بیضه، اختلالات روان‌پزشکی و رفتاری همچنین مشکلات دیگر در بدن انسان در هر دو جنس می‌باشد. هدف از پژوهش حاضر بررسی اثر یک دوره خود تزریقی سوستانون و مصرف امگا 3 بر سطوح سرمی کلسترول تام، عامل مغذی عصبی مشتق از مغز، لیپوپروتئین پر چگال، لیپوپروتئین کم چگال و آلانین آمینو ترانسفراز در بدنسازان مرد بود. مواد و روش‌ها: در این مطالعه نیمه تجربی، تعداد 14 مرد بدنساز (سن: 42/5±27/28 سال، وزن: 19/10±27/82 کیلوگرم، قد: 73/4±07/180 سانتی‌متر) به‌طور داوطلبانه وارد مطالعه شدند و به‌طور تصادفی به دو گروه تجربی 1 (7 نفر، مصرف سوستانون) و تجربی 2 (7 نفر، مصرف سوستانون و امگا 3) تقسیم شدند. همه آزمودنی‌ها به مدت 35 روز سوستانون تزریق کردند. در خلال این دوره، اعضاي گروه 2 روزانه به‌مدت 30 روز یک قرص امگا 3 (3 گرمی) مصرف کردند. در طول دوره‌ی مصرف، تمامی آزمودنی‌ها دارای تمرینات منظم بدنسازی بودند. نمونه‌های خونی در حالت ناشتا، 48 ساعت پیش و پس از دوره برای اندازه‌گیری سطوح کلسترول تام، عامل مغذی عصبی مشتق از مغز، لیپوپروتئین پر چگال، لیپوپروتئین کم چگال و آلانین آمینو ترانسفراز سرمی از آزمودنی‌ها گرفته شد. برای تجزیه و تحلیل داده‌ها از آزمون‌های t زوجی و تجزیه و تحلیل کوواریانس، استفاده و سطح معنی‌داری 05/0>P در نظر گرفته شد. نتایج: نتایج آزمون t زوجی نشان داد که در گروه 1 سطوح سرمی عامل مغذی عصبی مشتق از مغز آلانین آمینو ترانسفراز به‌طور معناداری افزایش یافت (05/0>P). اما در گروه 2 سطوح سرمی عامل مغذی عصبی مشتق از مغز و لیپوپروتئین پر چگال افزایش معنادار (05/0>P) و لیپوپروتئین كم چگال کاهش معنادار داشت (03/0=P). همچنین، نتایج آزمون کوواریانس نشان داد که اختلاف بین دو گروه تنها در غلظت‌های سرمی عامل مغذی عصبی مشتق از مغز معنادار شد (015/0=P) اما در متغیرهای دیگر معنادار نشد (05/0<P). نتیجه‌گیری: مصرف سوستانون به تنهایی و سوستانون به همراه امگا 3 به‌مدت 35 روز در بدنسازان مرد باعث افزایش سطوح سرمی عامل مغذی عصبی مشتق از مغز شد، اما مصرف همزمان سوستانون و امگا 3 اثرات بیشتری داشت.

Title: بررسی تمرینات ترکیبی کوتاه مدت بر بیان ژن‌های سرکوبگر تومور P53 و P21 در مردان مبتلا‌به سرطان پروستات

http://knh.shmu.ac.ir/index.php/site/article/view/1861

مقدمه: سرطان پروستات یکی از مهم‌ترین علل مرگ و میر ناشی از سرطان در مردان است. وجود پروتئین‌های سرکوبگر تومور یکی از مکانیسم‌های تنظیمی در کنترل پیشرفت این بیماری است. هدف از پژوهش حاضر بررسی تمرینات ترکیبی کوتاه مدت بر بیان ژن‌های سرکوبگر تومور P53 و P21 در مردان مبتلا‌به سرطان پروستات از نوع آدنوکارسینوما می‌باشد. مواد و روش‌ها: در این مطالعه نیمه تجربی، با مراجعه به بخش پاتولوژی بیمارستان فوق تخصصی بقیه الله الاعظم (عج) تهران و بررسی مدارک پزشکی مردان مبتلا‌به سرطان پروستات در شش ماه اول سال 1395 تعداد بیست مرد مبتلا‌به سرطان پروستات از نوع آدنوکارسینوما در مرحله T1 و T2 با میانگین سنی 71/7 ±60/62 و شاخص توده بدنی 40/3 ± 10/26 به‌صورت تصادفی انتخاب و به دو گروه کنترل (10=n) و تجربی (10=n) تقسیم شدند. گروه تجربی تمرینات ترکیبی را به مدت هشت هفته و هر هفته به مدت سه روز شامل تمرین مقاومتی با 60 الی 75 درصد یک تکرار بیشینه و تمرین هوازی با 60 الی 75 درصد حداکثر ضربان قلب انجام دادند. بیان ژن‌های P53 و P21 با روش Real-Time PCR اندازه گیری و به روش آماری t زوجی و مستقل مورد سنجش قرار گرفت. نتايج: بررسی نتایج حاصل نشان داد که تمرینات ترکیبی کوتاه مدت موجب افزایش معنی‌داری در بیان ژن P53 (000/0=P) و P21 (00/0=P) در گروه تجربی و نیز در مقایسه با گروه کنترل (00/0=P) گردید. بیان ژن P21 و P53 در گروه کنترل تفاوت معنی‌داری نداشت (05/0≤P). نتیجه‌گیری: افزایش معنی‌دار در توان هوازی و قدرت عضلانی متعاقب تمرینات ترکیبی کوتاه مدت و پیامد آن افزایش معنی‌دار در پروتئین‌های سرکوبگر تومور احتمالاً می‌تواند کاهش پیشرفت تومورهای سرطانی پروستات از نوع آدنوکارسینوما را موجب گردد.

Title: تأثیر تمرینات ترکیبی همزمان با مصرف دو مکمل امگا-3 وال- کارنتین بر سطح سرمی آیریزین، مقاومت به انسولین و نیمرخ لیپیدی در زنان دیابتی نوع 2

http://knh.shmu.ac.ir/index.php/site/article/view/1747

مقدمه: تعیین تأثیر تمرینات ترکیبی همزمان با مصرف دو مکمل امگا-3 وال- کارنتین بر سطح سرمی آیریزین، مقاومت به انسولین و نیمرخ لیپیدی در زنان دیابتی نوع 2 بود. مواد و روش‌ها: در این پژوهش که از نوع کارآزمایی بالینی بود، 58 زن داوطلب مبتلابه دیابت نوع 2 به‌صورت تصادفی به 6 گروه 10 نفری: 1- تمرین+دارونما، 2- تمرین+ مکمل امگا3، 3- تمرین+ مکمل ال کارنتین، 4- تمرین+ مکمل امگا3+ ال کارنتین، 5- مکمل امگا3+ ال کارنتین و 6- کنترل(8نفر) تقسیم شدند. تمرینات ترکیبی (8 هفته، 3 جلسه در هفته) شامل 30 دقیقه تمرین هوازی دویدن روی تردمیل و 40-30 دقیقه تمرین مقاومتی دایره‌ای بود. گروه امگا3 روزانه 2 عدد کپسول (1000 میلی‌گرم) امگا 3 و گروه ال –کارنتین 500 میلی‌گرم ال – کارنتین مصرف کردند. ازآزمودنی‌ها رضایت‌نامه کتبی، پرسشنامه سلامت عمومی و پرسشنامه فعالیت بدنی (PAR-Q) اخذ شد. نتایج در نرم‌افزار SPSS مورد تحلیل قرار گرفت. نتایج: یافته‌ها در زنان با میانگین سنی 1/6±7/52 (سال) بررسی و سطح سرمی آیریزین در تحلیل کوواریانس در گروه 2 (02/0P=) و 4(04/0(P= افزایش معناداری داشت. افزایش معنادار HDL را در گروه‌های 4 و 5 (001/0P=) و کاهش معنادار TG را در گروه 2 (01/0P=) و 4 (03/0=(P و گروه 5 (01/0(P= نشان داد. مقاومت به انسولین درگروه 2 و 5 )03/0=P) تنها در t همبسته کاهش معنی‌داری داشت. نتيجه‌گيري: یافته‌ها نشان دادند تمرین با مصرف مکمل امگا3 و تمرین با امگا 3 + ال کارنتین نسبت‌به دیگر گروه‌ها محرک مناسب‌تری برای افزایش سطح سرمی آیریزین و در نتیجه کاهش عوامل خطر دیابت هستند.

Title: مقایسه بیان ژن VEGF-A در پولیپ‌های آدنومای کولون و رکتوم

http://knh.shmu.ac.ir/index.php/site/article/view/1825

مقدمه: سرطان روده بزرگ اغلب از پولیپ‌های آدنوما منشأ گرفته می‌شود. بررسی میزان بیان ژن‌هایی که در رگزایی نقش دارند می‌تواند به‌عنوان عاملی برای تشخیص مولکولی سیرپیشرفت پولیپ به سمت بدخیمی‌ باشد. هدف از این مطالعه مقایسه بیان ژن VEGF-A در پولیپ‌های آدنومای کولون و رکتوم و ارتباط آن با بروز بدخیمی می‌باشد. مواد و روش‌ها: در این مطالعه 50 نمونه از بیوپسی بیماران دارای پولیپ آدنومای روده بزرگ و 20 نمونه طبیعی افراد سالم از بیمارستان طالقانی تهران تهیه شد. اطلاعات بالینی بیمار ان جمع‌آوری گردید. استخراج RNA و cDNA سازی نمونه‌ها صورت گرفت. بررسی میزان بیان با استفاده از روشReal time PCR و کمیت‌سنجی مطلق توسط دستگاه 7500ABI سنجیده شد. از روش (∆∆ct- 2) جهت بررسی تغییرات بیان ژن بهره گرفته شد. یافته‌ها با استفاده از نرم‌افزار آماری Prism تجزیه و تحلیل گردید. نتایج: میزان بیان ژن VEGF-A در پولیپ‌های آدنوما نسبت‌به گروه کنترل افزایش‌یافته بود اما دارای تفاوت معنادار نبود( 05/0<P) و در پولیپ‌های آدنومای ناحیه کولون بالاتر از رکتوم بوده اما این تفاوت از لحاظ آماری معنادار نبود (05/0<P). نتیجه‌گیری: در میزان در مطالعه حاضر افزایش محسوس بیان ژن VEGF-A در نمونه‌ها (مقدار P بسیار نزدیک به معنادار بودن) مشاهده شد. از آنجایی که پولیپ‌های آدنوما پتانسیل بدخیمی بیشتری برای ایجاد بدخیمی دارند و افزایش بیان ژن VEGF-A در این نوع پولیپ‌ها ممکن است نقش مهمی در ایجاد این روند داشته باشد. همچنین محل قرارگیری پولیپ‌های آدنوما می‌تواند در میزان بیان ژن VEGF-A مؤثر باشد.
